# Healthcare without borders: A cross-sectional study of immigrant and nonimmigrant children admitted to a large public sector hospital in the Gauteng Province of South Africa

**DOI:** 10.1371/journal.pmed.1003565

**Published:** 2021-03-23

**Authors:** Gerhard H. Janse van Rensburg, Ute Dagmar Feucht, Jennifer Makin, Nanya le Clus, Theunis Avenant

**Affiliations:** 1 Department of Paediatrics, Kalafong Provincial Tertiary Hospital and University of Pretoria, Pretoria, South Africa; 2 District Clinical Specialist Team, Tshwane District Health Services, Gauteng Department of Health, Pretoria, South Africa; 3 Research Centre for Maternal, Fetal, Newborn and Child Health Care Strategies, University of Pretoria, Pretoria, South Africa; 4 Maternal and Infant Health Care Strategies Unit, Medical Research Council, Pretoria, South Africa

## Abstract

**Background:**

Human migration is a worldwide phenomenon that receives considerable attention from the media and healthcare authorities alike. A significant proportion of children seen at public sector health facilities in South Africa (SA) are immigrants, and gaps have previously been noted in their healthcare provision.

The objective of the study was to describe the characteristics and differences between the immigrant and SA children admitted to Kalafong Provincial Tertiary Hospital (KPTH), a large public sector hospital in the urban Gauteng Province of SA.

**Methods and findings:**

A cross-sectional study was conducted over a 4-month period during 2016 to 2017. Information was obtained through a structured questionnaire and health record review. The enrolled study participants included 508 children divided into 2 groups, namely 271 general paediatric patients and 237 neonates. Twenty-five percent of children in the neonatal group and 22.5% in the general paediatric group were immigrants. The parents/caregivers of the immigrant group had a lower educational level (*p* < 0.0001 neonatal and paediatric), lower income (neonatal *p* < 0.001; paediatric *p* = 0.024), difficulty communicating in English (*p* < 0.001 neonatal and paediatric), and were more likely residing in informal settlements (neonatal *p* = 0.001; paediatric *p* = 0.007) compared to the SA group. In the neonatal group, there was no difference in the number of antenatal care (ANC) visits, type of delivery, gestational age, and birth weight. In the general paediatric group, there was no difference in immunisation and vitamin A supplementation coverage, but when comparing growth, the immigrant group had more malnutrition compared to the SA group (*p* = 0.029 for wasting). There was no difference in the prevalence of maternal human immunodeficiency virus (HIV) infection, with equally good prevention of mother-to-child transmission (PMTCT) coverage. There was also no difference in reported difficulties by immigrants in terms of access to healthcare (neonatal *p* = 0.379; paediatric *p* = 0.246), although a large proportion (10%) of the neonates of immigrant mothers were born outside a medical facility.

**Conclusions:**

Although there were health-related differences between immigrant and SA children accessing in-hospital care, these were fewer than expected. Differences were found in parental educational level and socioeconomic factors, but these did not significantly affect ANC attendance, delivery outcomes, immunisation coverage, HIV prevalence, or PMTCT coverage. The immigrant population should be viewed as a high-risk group, with potential problems including suboptimal child growth. Health workers should advocate for all children in the community they are serving and promote tolerance, respect, and equal healthcare access.

## Author summary

**Why was this study done?:**

- Cross-border migration has continually increased in South Africa (SA), resulting in increased attention of health-related issues pertaining to immigrants from governmental organisations, the media, and communities themselves.
- Immigrant children presenting to public healthcare facilities in SA were thought to be worse off, compared to the local population, with regard to health-related issues.

**What did the researchers do and find?:**

- The characteristics and differences between the immigrant and SA children admitted to a large public sector hospital in an urban setting were investigated.
- Major differences between the 2 groups were found in parental education, income, housing environment, English language proficiency, and childhood malnutrition.
- Access to maternal and child healthcare was similar between the groups.

**What do these findings mean?:**

- Although fewer than expected health-related differences were found, the immigrant population should still be viewed as a high-risk group, with potential problems.
- Health workers should advocate for all children in the community they are serving and promote tolerance, respect, and equal healthcare access.

## Introduction

The number of international migrants reached 272 million worldwide in 2019, reflecting a 51% increase since 2010 [1]. Cross-border migration has continually increased in South Africa (SA) in the recent past due to available social infrastructure, educational opportunities, medical infrastructure, as well as political instability in neighbouring countries [2]. This has resulted in increased attention of issues pertaining to immigrants from SA governmental organisations, the media, and communities themselves.

Immigrants are known to be potentially marginalised and vulnerable [3]. Migrants in SA face many social and psychological challenges, as demonstrated by the xenophobic violence during 2008, which resulted in numerous deaths, injuries, and displacement of more than 100,000 immigrants [4]. More recently, in March 2018, the SA Human Rights Commission reported on public hospitals in the Gauteng Province denying migrants healthcare services linked to their nationality and/or legal status [5].

The SA Children’s Act of 2005 aims to protect all children in the country, irrespective of migration status, therefore ensuring legislated protection of migrant and refugee children. However, there may be confusion as to the rights of the migrant child, as the section of the bill pertaining to migrant children was initially included in the draft bill but later removed. Despite the Act not specifically mentioning migrant children, SA is a signatory to many United Nations conventions, including the Convention on the Rights of the Child, which provides for equal statutes to all children irrespective of their nationality. This Convention requires the State to act appropriately to promote the inherent right to life and the child’s right to development and survival, as well as to protect all children from all forms of discrimination and maltreatment [6].

Under SA law, however, doctors are required to determine the immigration status of patients before administering medical care. If the patient is deemed an illegal immigrant, the doctor has a legal duty to report this to the relevant authorities [7].

The international migrant population potentially encounters many barriers in accessing healthcare in SA, such as the denial of medical treatment based on the inability to produce the necessary documentation when attending healthcare facilities [8]. Language and communication barriers, as well as unfamiliar healthcare services and fear of deportation, all play a crucial role in creating barriers to healthcare access. The use of qualified interpreters, often unavailable in the SA healthcare setting, is necessary for adequate care of immigrant children, as lack of language support has been identified as a common barrier to healthcare success with an increased risk of medical errors and decreased patient satisfaction [9,10].

Immigrant children may have preexisting health problems, including a potentially high prevalence of infectious diseases, post-traumatic stress disorder, depression, and anxiety [9]. Migrants are also at risk of malnutrition, growth retardation, and developmental delay associated with poor nutrition and other causes [11]. Resettlement experiences may negatively impact important stages of development (physical, intellectual, social, and emotional), as outlined by a report from the United Nations High Commissioner for Refugees [12]. Immigrant families may also be more vulnerable to mental health problems. Many of these families face separation, with some of the siblings, or even parents, not living with them or not even residing in the same province or country, potentially leading to high levels of stress and anxiety [10]. This, combined with fear and discrimination in their residing community, can also exacerbate feelings of isolation and lead to mental health problems [10].

Immunisation status of immigrant children should be determined, and catch-up immunisation provided, as many of these children may have had limited access to vaccination [9]. In many cases, immunisation records will be lost. Other factors that need to be taken into consideration when deciding on which immunisations are needed are possible incomplete or missing health records or severe malnutrition at the time of immunisation, which could impair adequate immune response [9,11]. Immigrants and refugees may import infectious diseases, and many old and new diseases may emerge or reemerge because of immigration. Therefore, immigrant children should be screened for infectious diseases, although this requires knowledge of the disease patterns in the country of origin [3].

Kalafong Provincial Tertiary Hospital (KPTH) is a 760-bed public sector hospital, located in South Western Tshwane in the urban Gauteng Province of SA, within a metropolitan area with high migration. Patients served by the hospital come from diverse socioeconomic backgrounds including established suburbs as well as people living in informal settlements. Children admitted for in-hospital care to KPTH include a large percentage of immigrants who are generally perceived by local health workers to differ in terms of their health status and access to healthcare. Immigrants from a wide variety of countries are seen at KPTH. Although mostly from Africa (Zimbabwe, Malawi, Mozambique, Nigeria, Lesotho, Tanzania, Angola, and Swaziland), there are immigrants from far afield countries such as India and Pakistan. Immigrants that present to public healthcare facilities in SA are thought to be worse off, compared to the local population, with regard to severity of illness at presentation to health facilities, and other health-related issues such as suboptimal growth, development, and immunisation coverage, thought to be attributed to level of education, lower income, language barriers, and hurdles in terms of access to care. This formed the basis of our hypothesis. The primary aim of this study was to document the health status of children admitted to KPTH and to identify differences between the immigrant and nonimmigrant groups in order to potentially inform future policy decisions.

## Methods and findings

A cross-sectional descriptive study was conducted in 2 phases over a study period of 4 months (2016 to 2017) at KPTH. Before the study was conducted, the study protocol was submitted and approved by the research review board and relevant institutional ethics committee. Children, younger than 13 years, who were admitted to the paediatric and neonatal wards during this period were enrolled in the study. Children were excluded if no parental/caregiver consent could be obtained or if they were discharged before study enrolment could occur. Immigrants were under no circumstances denied emergency medical care based on their legal standing or immigration status. The aim of this study was not to identify the legal standing of immigrants, as this would have created an ethical dilemma for the study investigators. The delivery of medical care was also not dependent on participation in the study. The questionnaires were administered by study investigators (doctors working in the paediatric department) during the patient’s period of hospital stay, after initial rapport and a trust relationship had been established with the parent/caregiver. The questionnaire was conducted orally by the study investigators, and although the questionnaire was in English, the study investigators translated it into a language that was comprehensible to the parent/caregiver. This was not always the person’s first language but would be one that the parent/caregiver could understand and speak. Where communication was difficult, an alternate investigator or an interpreter was used.

Some demographic information like race/ethnicity was not documented in this study due to stigmatisation risks, but it should be noted that the larger proportion of patients admitted to KPTH are black South Africans, which corresponds with the national statistics. Participants were enrolled in 2 groups, a neonatal group and a general paediatric group. The neonatal group consisted of patients, either inborn or outborn, who were less than 4 weeks of age. The paediatric group included patients older than 1 month and younger than 13 years admitted to KPTH. When comparing variables between the SA and immigrant group, we compared the SA mother and foreign mother/caregiver. By law, a child is considered a SA citizen if either of the parents are South African or have permanent residency.

A detailed demographic, social, and clinical history was obtained using a structured questionnaire (S1 Questionnaire). We further documented aspects related to maternal and child health, including access to antenatal care (ANC), birth history, maternal human immunodeficiency virus (HIV) status as well as prevention of mother-to-child transmission (PMTCT) interventions, as outlined by SA national guidelines. We evaluated infant feeding practices, immunisation coverage (assessed as missed immunisations whether using the SA or country of origin immunisation schedule), and ease of healthcare access. This included participants being asked about access to care problems, such as refusal of healthcare based on nationality, immigration status, documentation problems regarding nationality and citizenship, high unaffordable consultation fees for foreigners, communication problems between patient and healthcare personnel, xenophobic comments, verbal insults, discriminatory behaviour from medical personnel, long waiting times, medicine shortages, and transport problems.

The study was approved by the Faculty of Health Sciences Research Ethics Committee of the University of Pretoria as well as the KPTH Ethics Committee. Confidentiality was considered of the utmost importance, and all efforts were made to ensure that this vulnerable group of patients were not discriminated against because of the study, including use of unique study numbers as well as use of private rooms in the hospital wards for conducting the interview, wherever possible.

A sample size calculation was not possible as there was no source of information on the rate of admission of migrants to either the neonatal or paediatric wards. In addition, sample size calculations were hampered by the wide range of areas probed such as HIV, tuberculosis (TB), and nutritional status. It was thus decided, based on practical reasons, that the data would be collected over 4 months. This study utilised non-probability convenience sampling and was deemed exploratory. Missing data were not dealt with specifically, other than treating it with caution if there were more than 20% data missing from any variable.

Data analysis was performed using SPSS V23. Descriptive statistics were used to describe the maternal and patient characteristics. Means and standard deviations were calculated for continuous normally distributed data, while medians and ranges were used for non-normally distributed or ordinal data. Normality of the distribution was established making use of histograms and the Shapiro–Wilk test for normality. In the case of categorical data, frequencies and percentages were used. When comparing the immigrant and nonimmigrant groups, 2-sided *t* tests for independent samples were used in the case of continuous normally distributed data and a Mann–Whitney U (MWU) test for non-normally distributed or ordinal data. Mean differences and confidence intervals (CIs) were calculated for normally distributed data. Categorical variables were examined using 2 sided chi-squared or Fisher exact tests where the expected values in any of the cells was below 5. The Fisher Freeman Halton Exact (FFHE) test was used in the case of C2XR>1 tables. Odds ratios (ORs) and CIs were calculated for 2 × 2 contingency tables. A *p*-value of ≤0.05 was considered significant.

### Neonatal group

**Fig 1:**
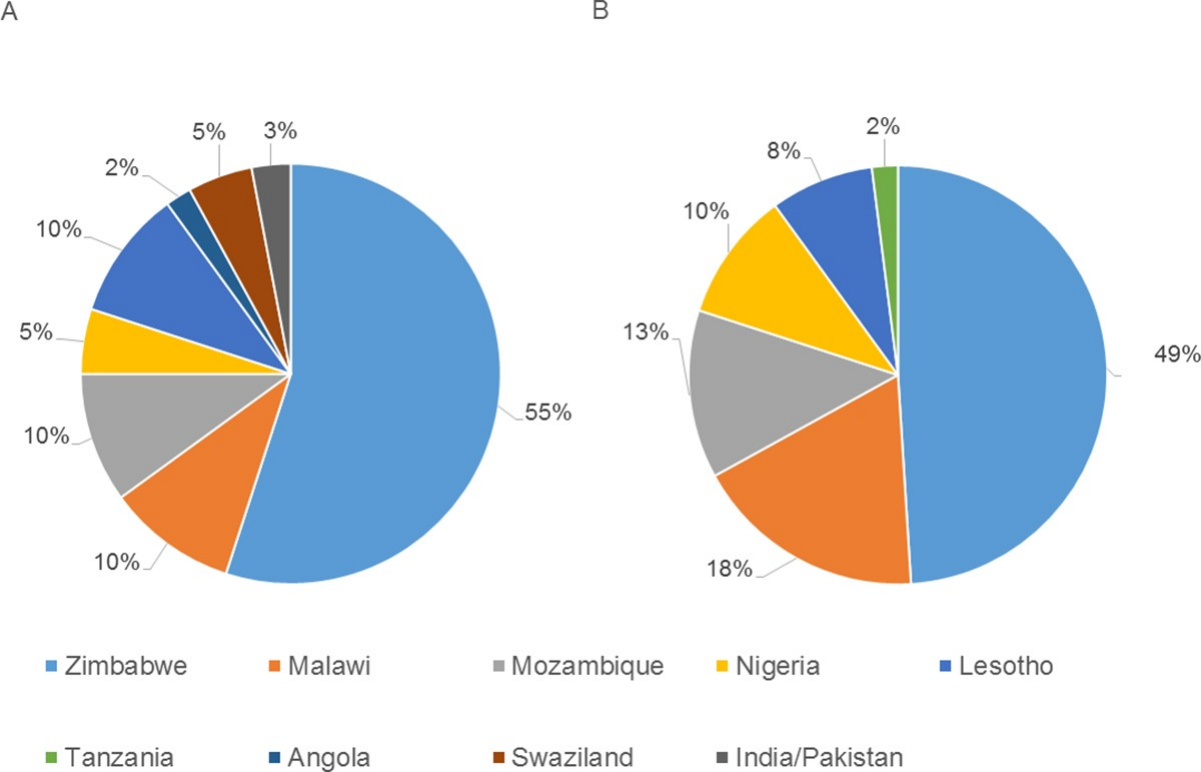
Countries of origin of foreign parents/caregivers. (A) Neonatal group (*n* = 60). (B) General paediatric group (*n* = 61).

Among neonatal admissions, 25.3% were babies to immigrant mothers, with the majority from neighbouring countries like Zimbabwe (55%) and Mozambique (10%) (Fig 1). Immigrant mothers were on average younger than the SA group (26.6 years versus 30.8 years; *p* < 0.0001) (Table 1). The immigrant group faced many socioeconomic challenges when compared to the SA group. They had a lower educational level (*p* < 0.0001) and income (*p* < 0.001) and the majority (66.1% versus 40.6%; OR 0.4, 95% CI: 0.2 to 0.6) resided in informal settlements, with fewer children in the family structure.

**Table 1:** General characteristics of the neonatal group.

Characteristics			South African *(n* = 177)	Immigrant (*n* = 60)	*p*-value	OR (CI)	MD (CI)
**Total**	*n* (%)	237	177 (74.7)	60 (25.3)			
**Twins**	*n* (%)		8 (4.5)	5 (8.3)			
**Maternal age (years)**	Mean (SD)		30.8 (6.6)	26.6 (5.7)	<0.0001		3.5 (1.6, 5.4)
**Paternal age (years)**	Mean (SD)		33.9 (7.4)	30.7 (6.2)	0.004		3.2 (1.0, 5.3)
**Length of stay in hospital (days)**	Mean (SD)		11.4 (14.4)	11.6 (12.6)	0.957		
**Poor English proficiency**	*n* (%)		18 (10.2)	22 (37.3)	<0.0001	5.3 (2.6–10.8)	
**Number of ANC visits**	Mean (SD)		4.5 (2.6)	4.6 (2.8)	0.851		−0.08 (−0.87, 0.71)
**Cesarean delivery**	*n* (%)		99 (53.3)	29 (44.6)	0.217	1.4 (0.8–2.5)	
**Gestational age (weeks)**	Mean (SD)		35.5 (3.9)	35.4 (4.5)	0.903		0.7 (−1.1, 1.3)
**Birth weight (kg)**	Mean (SD)		2.34 (0.88)	2.33 (0.87)	0.926		0.01 (−0.24, 0.27)
**HIV–positive mothers**	*n* (%)		50 (28.2)	10 (16.7)	0.078	1.9 (0.9–4.3)	
**PMTCT, mother–infant received treatment**	*n* (%)	Yes No	44 (89.8) 5 (10.2)	9 (90.0) 1 (10.0)	0.984	0.98 (0.10–9.4)	
		Unknown	1(0.0)				
**Syphilis serology (mother)**	*n* (%)	Negative Positive	165 (100.0) 0 (0.0)	54 (98.2) 1 (1.8)	0.99		
**Difficulty attending ANC**	*n* (%)	Yes	11 (6.2)	5 (8.3)	0.386	0.8 (0.3, 2.3)*	
		No	161 (91.0)	55 (91.7)			
		Did not attend	5 (2.8)	0 (0.0)			
**Place of delivery**	*n* (%)	Hospital	167 (94.4)	52 (86.7)			
		Clinic	5 (2.8)	2 (3.3)	0.054		
		Born outside of hospital	5 (2.8)	6 (10)			
**Breastfeeding**	*n* (%)	Yes	169 (97.7)	61(100.0)	0.575		
		No	4 (2.3)	0 (0.0)			

* This is the result if one leaves out the category did not attend—if that is included, then no OR can be calculated.

ANC, antenatal care; CI, confidence interval; HIV, human immunodeficiency virus; MD, mean difference; OR, odds ratio; PMTCT, prevention of mother-to-child transmission; SD, standard deviation.

The difference between paternal employment rate of the 2 groups (SA versus immigrant) was not statistically significant, but the opposite was true with regard to the mothers, with more immigrant mothers being unemployed (52.8% versus 86.2%; OR 5.6, 95% CI: 2.5 to 12.4). A larger proportion of the immigrant mothers could not communicate well in English [37.3% versus 10.2%; *p* < 0.0001] (Tables 1 and 2). There was no difference in the number of ANC visits, type of delivery, gestational age, and birth weight (Table 1).

**Table 2:** Socioeconomic factors of the neonatal group.

Characteristics			South African *(n* = 177)	Immigrant *(n* = 60)	*p*-value	OR (CI)	MD (CI)
**Father employed**	*n* (%)	Yes	145 (82.9)	53 (91.4)	0.115	0.5 (0.2, 1.2)	
		No	30 (17.1)	5 (8.6)			
**Mother employed**	*n* (%)	Yes	83 (47.2)	8 (13.8)	<0.0001	5.6 (2.5, 12.4)	
		No	93 (52.8)	50 (86.2)			
**Mother education**	*n* (%)	No school	1 (0.6)	3 (5.1)			
		Grade 1–7	6 (3.4)	8 (13.6)	<0.0001^@^		
		Grade 8–10	59 (33.3)	33 (55.9)			
		Grade 11–12	66 (37.3)	12 (20.3)			
		Tertiary	45 (25.4)	3 (5.1)			
**Father education**	*n* (%)	No school	1 (0.6)	1 (2.0)	<0.0001^@^		
		Grade 1–7	4 (2.5)	4 (8.0)			
		Grade 8–10	33 (20.2)	21 (42.0)			
		Grade 11–12	81 (49.7)	18 (36.0)			
		Tertiary	44 (27.0)	6 (12.0)			
**Number of children in family structure locally**	Mean (SD)	Mean (SD)	1.5 (1.4)	1.1 (1.8)	0.039		0.4 (0.2, 0.8)
**Income**	*n* (%)	≤ ZAR 2,500	37 (22.6)	30 (53.6)			
		> ZAR 2,500	39 (23.8)	15 (26.8)	<0.001^@^		
		> ZAR 5,000	48 (29.3)	5 (8.9)			
		> ZAR 10,000	25 (15.2)	3 (5.4)			
		> ZAR 20,000	12 (7.3)	2 (3.6)			
		> ZAR 30,000	3 (1.8)	1 (1.8)			
**Housing type**	*n* (%)	Informal	71 (40.6)	39 (66.1)	0.001	0.4 (0.2, 0.6)	
		Formal	104 (59.4)	20 (33.9)			
**Self-reported difficulty in access to healthcare**	*n* (%)	Yes	22 (12.6)	10 (17.2)	0.379	0.7 (0.3, 1.6)	
		No	152 (87.4)	48 (82.8)			
**Anthropometry**^**+**^	Median (range)	Weight-for-age Z-score	−0.68 (−3.6, 4.6)	−0.69 (−3.0, 2.3)	0.725*		
		Height-for-age Z-score	0.40 (−5.6, 7.0)	0.27 (−2.6, 3.0)	0.858*		
		Head circumference-for-age Z-score	0.90 (−3.7, 9.9)	0.30 (−2.2, 3.4)	0.775*		

^+^ WHO growth standards were used as reference.

* MWU test.

^@^ FFHE test.

^#^ ZAR = South African Rand (1 ZAR = $0.079 USD on December 31, 2016).

CI, confidence interval; FFHE, Fisher Freeman Halton Exact; MD, mean difference; MWU, Mann–Whitney U; OR, odds ratio; SD, standard deviation; WHO, World Health Organization.

The SA group had a higher percentage of HIV–positive mothers (28.2% versus 16.7%; OR 1.9, 95% CI: 0.9 to 4.3), but statistical testing failed to show a difference between the 2 groups (*p* = 0.078). Close to 90% of mother–infant pairs in both groups received appropriate antiretroviral drugs for PMTCT. In some instances, the mother was diagnosed and never initiated on antiretroviral therapy (ART) or was initiated but defaulted treatment. In line with national policy, breastfeeding was the preferred method of infant feeding in both groups (97.7% [SA] versus 100% [immigrant]).

Self-reported difficulty in access to healthcare was not significantly different between the 2 groups (*p* = 0.379); however, a large percentage of the immigrant mothers delivered their babies outside a healthcare facility (10% versus 2.8%; *p* = 0.054).

### Paediatric group

In the general paediatric group, a total of 271 patients were enrolled of which 210 (77.5%) were from the SA group and 61 (22.5%) from the immigrant group. Similar to the neonatal group, most immigrants were from Zimbabwe (49%), followed by Malawi (18%) and then Mozambique (13%) (Fig 1). The mean age of the mothers (29.8 years [SA] versus 29.2 years [immigrant]; *p* = 0.562) and fathers (34.3 years [SA] versus 34.0 years [immigrant]; *p* = 0.773) were similar (Table 3).

**Table 3:** General characteristics of general paediatric group.

Characteristics			South African (*n* = 210)	Immigrant (*n* = 61)	*p*-value	OR (CI)	MD (CI)
**Maternal age (years)**		Mean (SD)	29.8 (7.0)	29.2 (6.1)	0.562		0.6 (−1.4, 2.7)
**Paternal age (years)**		Mean (SD)	34.3 (7.5)	34.0 (7.5)	0.773		0.3 (−2.0, 2.7)
**Length of hospital stay (days)**		Mean (SD)	5.3 (12.8)	4.8 (4.7)	0.794		0.5 (−3.2, 4.2)
**Poor English proficiency**		*n* (%)	10 (4.6)	13 (25.0)	<0.0001	0.14 (0.06, 0.4)	
**HIV–positive child**		*n* (%)	14 (6.4)	5 (9.6)	0.606	0.6 (0.2, 1.9)	
**PMTCT treatment to child**	Yes	*n* (%)	55 (25.2)	14 (26.9)			
	No		163 (74.4)	38 (73.1)	0.801	0.9 (0.5, 1.8)	
	Not recorded		1 (0.5)	0			
**TB-infected child**	Yes	*n* (%)	7 (3.3)	2 (3.8)			
	No		205 (96.7)	50 (96.2)	0.846	0.9 (0.2, 4.2)	
**Exclusively breastfed for 6 months**	Yes	*n* (%)	110 (57.3)	34 (72.3)			
	No		78 (40.6)	12 (25.5)	0.126		
	Exclusive formula		4 (2.1)	1 (2.1)			
**Immunisation schedule up to date**	Yes	*n* (%)	156 (71.2)	33 (63.5)			
	No		40 (18.3)	10 (19.2)	0.362^@^		
	Not recorded		23 (10.5)	9 (17.3)			
**Vitamin A supplementation up to date**	Yes	*n* (%)	138 (63.0)	34 (66.7)			
	No		28 (12.4)	5 (9.8)			
	Too young		17 (7.8)	4 (7.8)	0.960^@^		
	Unknown		36 (16.4)	8 (15.7)			

* MWU test.

^@^ FFHE test.

CI, confidence interval; FFHE, Fisher Freeman Halton Exact; MD, mean difference; MWU, Mann–Whitney U; OR, odds ratio; PMTCT, prevention of mother-to-child transmission; SD, standard deviation; TB, tuberculosis.

With regard to the immigrant group, the mean duration of paternal stay in SA was 74 months compared to 48 months of the mother. Both groups had a comparable family structure size locally (mean 2.1 versus 2.1; *p* = 0.933), which includes the number of children currently staying with the mother but excludes children left behind in the country of origin. Similar to the neonatal group, the mothers of the paediatric immigrant group had more difficulty communicating in English compared to local mothers (*p* < 0.0001). The immigrant group had a lower educational level with 31.8% achieving grade 11 or 12 and 11.4% with a tertiary education (*p* < 0.0001). Immigrants had a lower income, and everyone interviewed earned less than 10,000 ZAR/month (± $729 USD/month), with 58.8% earning ≤2,500 ZAR/month (± $182 USD/month) (*p* = 0.021), and they more often resided in informal housing (66.7% versus 45.8%; OR 0.4, 95% CI: 0.2 to 0.8). More immigrant fathers than South African fathers were employed (89.8% versus 73.7%; *p* = 0.016). In contrast, fewer immigrant mothers were employed (26.0% versus 42.9%; *p* = 0.028) (Table 4).

**Table 4:** Socioeconomic factors of the general paediatric group.

Characteristics			South African (*n* = 210)	Immigrant (*n* = 61)	*p*-value	OR (CI)	MD (CI)
**Father employed**	Yes	*n* (%)	151 (73.7)	44 (89.8)	0.016	0.3 (0.1, 0.8)	
	No		54 (26.3)	5 (10.2)			
**Mother employed**	Yes	*n* (%)	93 (42.9)	13 (26.0)	0.028	2.1 (1.1, 4.2)	
	No		124 (57.1)	37 (74.0)			
**Mother education**	No school	*n* (%)	1 (0.5)	0 (0)			
	Grade 1–7		5 (2.3)	10 (22.2)			
	Grade 8–10		77 (35.8)	23 (51.1)	<0.0001^@^		
	Grade 11–12		82 (38.1)	7 (15.6)			
	Tertiary		50 (23.3)	5 (11.1)			
			215	45			
**Father education**	No school	*n* (%)	2 (1.1)	3 (6.8)			
	Grade 1–7		1 (0.5)	7 (15.9)	<0.0001^@^		
	Grade 8–10		51 (27.3)	15 (34.1)			
	Grade 11–12		85 (45.5)	14 (31.8)			
	Tertiary		48 (25.7)	5 (11.4)			
**Number of children in family structure locally**		Mean (SD)	2.1 (1.2)	2.1 (1.2)	0.933		0.02 (−0.4, 0.4)
**Income**	≤ ZAR 2,500	*n* (%)	78 (37.9)	30 (58.8)			
	> ZAR 2,500		60 (29.1)	12 (23.5)			
	> ZAR 5,000		35 (17.0)	9 (17.6)	0.024^@^		
	> ZAR 10,000		19 (9.2)	0			
	> ZAR 20,000		8 (3.9)	0			
	> ZAR 30,000		6 (2.9)	0			
**Housing type**	Informal	*n* (%)	99 (45.8)	34 (66.7)		0.4 (0.2, 0.8)	
	Formal		117 (54.2)	17 (33.3)	0.007		
**Self-reported difficulty in access to healthcare**	Yes	*n* (%)	32 (14.6)	11 (21.2)		0.6 (0.3, 1.4)	
	No		187 (85.4)	41 (78.8)	0.246		
**Anthropometry**^**+**^	Weight-for-age Z-score	Median (range)	−0.98 (−7.0, 6.8)	−1.0 (−5.4, 2.1)	0.201*		
	Height-for-age Z-score		−0.66 (−8.6, 20.4)	−0.85 (−7.2, 4.4)	0.674*		
	BMI-for-age Z-score		−0.66 (−5.7, 3.4)	−1.30 (−4.5, 1.9)	0.029*		
	MUAC-for-age Z-score		−0.46 (−5.1, 2.7)	−0.84 (−3.8, 1.9)	0.118*		
	Head circumference-for-age Z-score		−0.20 (−4.6, 8.1)	0.31 (−3.5, 6.11)	0.869*		

^+^ WHO growth standards were used as reference.

* MWU test.

^@^ FFHE test.

^#^ ZAR = South African Rand (1 ZAR = $0.079 USD on December 31, 2016).

BMI, body mass index; CI, confidence interval; FFHE, Fisher Freeman Halton Exact; MD, mean difference; MUAC, mid upper arm circumference; MWU, Mann–Whitney U; OR, odds ratio; SD, standard deviation; WHO, World Health Organization.

There was no difference in the prevalence of TB and HIV between the 2 groups of children. PMTCT coverage and choice of infant feeding was also similar, and no difference in immunisation coverage and vitamin A administration rates were observed between the groups. When comparing growth according to age- and sex-adjusted standards (World Health Organization Z-scores), it was observed that a higher percentage of immigrant group suffered from malnutrition compared to the SA group (body mass index Z-scores (−0.66 versus −1.30) (*p* = 0.029).

A larger proportion of the SA group entered the healthcare system directly at hospital level in contrast to the immigrant group (44.7% versus 29.4%; *p* = 0.073). The majority of first visits for immigrants were at clinic level (56.9%). There was no difference in the proportion of cases with self-reported difficulties in access to care between the 2 groups (14.6% [SA] versus 21.2% [immigrant]; OR 0.6, 95% CI: 0.3 to 1.4).

## Discussion

This is the first SA paediatric study describing the demographics and examining the specific health-related concerns of immigrant children presenting to a public health facility. A large proportion of children who were admitted to KPTH were immigrants (25.3% neonates; 22.5% general paediatrics). The hypothesised differences between the SA and immigrant groups were however less significant than expected. Before this study was conducted, it was perceived that the immigrant group had difficulty in accessing healthcare, especially at clinic level. This was not confirmed, as more immigrants first consulted at a clinic before coming to hospital. This is in contrast with media reports stating the opposite [5,13]. Reported episodes of being denied access to care might be isolated to specific healthcare facilities and not be generalisable to the whole immigrant population. It may, however, reflect reporter bias with underreporting of access to care problems by the immigrant group, despite our best efforts to build rapport and ensure confidentiality during the interview process. It also needs to be taken into account that the study group consisted only of patients that were able to access the hospital. There could be many reasons for underreporting issues relating to access to care, including fear of prosecution, deportation, ostracisation, or discrimination in terms of medical care. Being said, all the patients whose parents/caregivers were interviewed in the study had already been admitted to hospital and were receiving medical treatment, which could negate the aforementioned problems.

Despite not finding crude differences in self-reported access to healthcare, the fact that a significant proportion of the immigrant group were not able to deliver in a healthcare facility is a reason for concern. On the one hand, it may be a proxy for unreported access to care problems or, on the other hand, might be related to socioeconomic factors, i.e., distance from hospital, transport problems, or being unaccustomed to the SA healthcare system. It is not clear whether the large proportion of the SA group who were accessing healthcare directly at hospital level constitutes appropriate behaviour or not, as this aspect was not assessed.

The longer mean duration of paternal versus maternal stay in SA likely indicates that the fathers immigrated to SA first, with the intent to find a home and employment. When comparing childhood growth, it showed that immigrant children suffered more from malnutrition compared to the SA group. A possible reason is that immigrant children may already be malnourished before arrival. The parents also have a lower earning potential compared to the SA group, and many immigrants do not qualify for social support in SA. This inverse relationship between socioeconomic status and malnutrition in sub-Saharan Africa is well known and has been described previously by Fotso and colleagues [14]. Added to these problems is English proficiency, which was found to be worse in the immigrant group and may be linked to the level of education. This too is a potential barrier when caregivers seek healthcare and employment.

No statistically significant differences were found between the 2 groups in terms of ANC visits and delivery outcomes, immunisation and vitamin A coverage, HIV prevalence, and PMTCT intervention program coverage. This stands in contrast with reports of difficulty in access to healthcare.

Findings related to the educational level, income, family structure, and housing relate to the group of immigrants that present to KPTH and does not encompass immigrants of a higher socioeconomic class who potentially attend private hospitals in the area. The fact that there was no significant difference in access to healthcare of the immigrant group, despite efforts to ensure confidentiality, may still reflect reporter bias as mentioned before. Language barriers seemingly did not influence the reporting of access to care problems. Interpreters were used whenever there were communication problems.

The results found in this study are potentially representative of the immigrant population in large South African metropolitan areas, especially those seeking healthcare in the public sector, although regional differences may exist in terms of the local healthcare authorities, regulations, and attitudes in dealing with immigrants at health facilities. Most parameters were not studied in depth as the study was mainly exploratory, designed to provide an overview of the current situation rather than a detailed analysis. A further limitation was the exclusion of patients who were discharged from the ward before they could be enrolled into the study. This might have led to bias.

South African doctors have a legal duty to report illegal immigrants to the authority [7]. Considering reports of cases where immigrants were denied healthcare, based on nationality and legal status, this law is clearly ethically problematic. Doctors should advocate for all children in the community they are serving and promote tolerance, respect, and equal healthcare access [9,15]. This apparent discordance between the Hippocratic Oath and professional obligations highlights the need for a discussion in the broader South African context, where migration from other African countries to SA is a prominent feature in the sociopolitical context, but with a need for a clarity in terms of the policies.

## Conclusions

Although there were differences demonstrated between immigrant and SA children accessing care in hospital, these were fewer than expected. The differences in parental educational level and socioeconomic factors did not appear to have impacted on ANC attendance, delivery outcomes, immunisation coverage, HIV prevalence, and PMTCT coverage. The malnutrition found in the paediatric immigrant population highlights the fact that this group should be viewed as a high-risk group, with potential growth deficits. This ought to inform policy decisions or emphasise the fact that SA and immigrant children should at least receive similar healthcare at healthcare facilities.

## Supporting information

S1 STROBE Checklist Checklist of items that should be included in reports of cross-sectional studies. (PDF)

S1 Questionnaire Data collection questionnaire. (DOCX)

## Abbreviations

ANC: antenatal care
ART: antiretroviral therapy
CI: confidence interval
FFHE: Fisher Freeman Halton Exact
HIV: human immunodeficiency virus
KPTH: Kalafong Provincial Tertiary Hospital
MWU: Mann–Whitney U
OR: odds ratio
PMTCT: prevention of mother-to-child transmission
SA: South Africa
TB: tuberculosis
