# Supplementary material for: Healthcare without borders: A cross-sectional study of immigrant and nonimmigrant children admitted to a large public sector hospital in the Gauteng Province of South Africa
Source: PLoS Med. 2021 Mar 23;18(3):e1003565. doi: 10.1371/journal.pmed.1003565 (PMC8023456; doi:10.1371/journal.pmed.1003565)
Supplement: S1 Questionnaire — (DOCX) [file pmed.1003565.s002.docx]

Appendix C **Questionnaire** NAME OF INTERVIEWER:_______________________

| Section A: Demographic Information |
| --- |

Patient’s particulars

Patient study number ____________________

Gender M / F

Date of Admission ____________________

Date of Birth ____________________

Place of Birth ____________________

Date of Discharge ____________________

File Number ____________________

Date of Death ____________________

Ward ____________________

Name of Person Interviewed ____________________

Interviewee relationship to Patient ______________

**Thank you very much for taking the time to talk to us. With this questionnaire we aim to better understand your circumstances and with your help try and improve health care for other children.**

**Firstly we will ask you a few questions so we can get to know you better.**

1. In which suburb/area does your/the child live? __________________________________________
2. To whom does the address belong?

FATHER□ - MOTHER□ - OTHER

1. What is your home language? ________________________________________________
2. Do you speak English?

| EXTREMELY WELL | GOOD | POORLY |
| --- | --- | --- |

Notes_________________________________________________________________________

| Section B: Neonatal Patients |
| --- |

**Next we would like to ask you a few questions about yourself and your baby.**

1. Ward admitted to:

| WARD 27 | WARD 28 | WARD 4A | NICU |
| --- | --- | --- | --- |

1. At how many weeks was your baby born?____________
2. Anthropometry at birth
3. BIRTH WEIGHT: _______ b. HEAD CIRCUMFERENCE: ________c. LENGTH: ________
4. Did you attend antenatal clinic when you were pregnant?  **Y / N**
5. If “**yes”**, how many visits? __________________
6. Where did you book antenatally? (eg. Clinic, Hospital, GP) __________________
7. Did you have any complications while you were pregnant? **Y / N**

If “**Yes**” provide detail:

1. Do you know your HIV status? **Y / N**
2. If yes, are you HIV positive: **Y / N**
3. Are you on treatment for HIV? **Y / N**
4. How long have you been on treatment? ________________________
5. Did you receive PMTCT (prevention of mother to child transmission) treatment during your pregnancy? **Y / N**

If “**Yes**” provide detail: What treatment did the mom receive and for how long?

1. Do you know your partner's HIV status? **Y / N**
2. Is your partner taking treatment for HIV? **Y / N / Unsure**
3. How long has your partner been on HIV treatment? ________________________
4. Have you been tested for syphilis? **Y / N/Does not know**

6.1. IF **YES**, RESULT ________________________

1. Did you encounter problems or difficulties when trying to attend antenatal clinic?

If “Yes” provide detail:

| Section C: All patients |
| --- |

**Next we would like to ask you a few questions about yourself and your child**

1. **Maternal contraception, pregnancy and birth**
2. What type of delivery did you have? (please tick)

| NVD | C/S |
| --- | --- |

1. Where did the delivery take place? (please tick)

| BORN OUTSIDE HEALTH CARE FACILITY | CLINIC | HOSPITAL |
| --- | --- | --- |

1. Was this a planned pregnancy? **Y / N**
2. Did you have access to contraception/birth control? **Y / N**

If “**No”**, why not or what difficulties did you have when trying to get contraception? Please elaborate.

1. **Anthropometry (**please supply absolute values)

| WEIGHT |  | MID UPPER ARM CIRCUMFERENCE |  |
| --- | --- | --- | --- |
| LENGTH |  | HEAD CIRCUMFERENCE |  |

1. **HIV**
2. Has your child been tested for HIV within the past **3 months**? **Y / N**
3. What is your child’s HIV result? ___________
4. Did you receive any PMTCT (prevention of mother to child transmission) treatment during your pregnancy? **Y / N**
5. Did your child receive any co-trimoxazole (Bactrim) treatment? **Y / N**
6. Is your child currently taking HIV treatment? **Y / N**
7. Last known viral load _________________ Date____________
8. Last CD4+ count _________________ Date____________
9. WHO Staging Criteria _________________
10. CDC Staging Criteria _________________
11. For how long has your child been on ART? _______________
12. Has your child ever stopped taking HIV treatment? **Y / N**

If “Yes” why

1. **Tuberculosis**
2. Has your child ever been treated for tuberculosis? Y / N

if **Yes**, when?________

1. For how long did your child take treatment for tuberculosis? ______________________
2. What treatment is your child taking?
3. Is there anyone at home that is currently being treated for tuberculosis? Please explain **who**, **how long** they have been on treatment for.
4. Did your child receive any treatment to prevent the child from getting TB? **Y / N**
5. If yes, for how long? _________
6. Is there anyone at home with symptoms of tuberculosis? (chronic cough, weight loss, loss of appetite, coughing blood, night sweats or fever)
7. **Breastfeeding**
   1. Have you ever breastfed your child? **Y / N**
   2. If **yes**, for how long? ___________
   3. **When** did you stop breastfeeding? ___________
   4. **Why** did you stop breastfeeding?
   5. Did you **exclusively** breastfeed for the first 6 months of your child's life? **Y / N**
   6. If **No**, **when** have you started giving extra food to your baby and what did you give as first food?
   7. Why did you give extra food except for breastmilk?
8. **Road to Health Booklet:**
9. Which country’s immunization schedule does your child follow?________________
10. Did your child receive all his/her immunizations as required by above schedule up to **now**: **Y / N**
11. Vit A received **Y/N**

Immunisations received:

| BCG |  |  |  |  |  |
| --- | --- | --- | --- | --- | --- |
| Polio |  |  |  |  |  |
| DTP/DTaP/Td |  |  |  |  |  |
| Hepatitis B |  |  |  |  |  |
| Hib |  |  |  |  |  |
| Measles |  |  |  |  |  |
| PCV |  |  |  |  |  |
| Rotavirus |  |  |  |  |  |
| Other  (Comment) |  |  |  |  |  |

1. Reasons for missing if applicable
2. Does your child have any other chronic health problems?
3. **Social:**
4. Are the parents working?

| Father | Y | N | Mother | Y | N | Caregiver 1 | Y | N | Caregiver 2 | Y | N |
| --- | --- | --- | --- | --- | --- | --- | --- | --- | --- | --- | --- |
| Notes: | | |  | | |  | | |  | | |

What is the relationship of the caregiver to the patient?

Caregiver 1: ____________________

Caregiver 2: ____________________

1. If the parents are working, who are the caregivers when the parents are working?
2. Maternal age: _____
3. What is the mother's highest level of education?

No School

| Gr.1  SubA | Gr.2  SubB | Gr.3  Std.1 | Gr.4  Std2 | Gr.5  Std3 | Gr.6 | Gr.7 | Gr.8 | Gr.9 | Gr.10 | Gr.11 | Gr.12 | Tech/College |
| --- | --- | --- | --- | --- | --- | --- | --- | --- | --- | --- | --- | --- |

1. Paternal age:
2. What is the father's highest level of education?

No School

| Gr.1  SubA | Gr.2  SubB | Gr.3  Std.1 | Gr.4  Std2 | Gr.5  Std3 | Gr.6 | Gr.7 | Gr.8 | Gr.9 | Gr.10 | Gr.11 | Gr.12 | Tech/College |
| --- | --- | --- | --- | --- | --- | --- | --- | --- | --- | --- | --- | --- |

What is the parents' relationship?

| Single | Co-habiting | Married* | Divorced | Widowed |
| --- | --- | --- | --- | --- |

*Includes traditional marriage

1. How many children in the current family structure? ________________________
2. What is their **sex** and **age**? Where do they **stay**?

| **Gender** | **Ages** | | | | | | |
| --- | --- | --- | --- | --- | --- | --- | --- |
| **Male** |  |  |  |  |  |  |  |
| Stay |  |  |  |  |  |  |  |
| **Female** |  |  |  |  |  |  |  |
| Stay |  |  |  |  |  |  |  |

1. With whom do they stay_________________
2. Who provides financially for the child?________________
3. What is your total household income per month:

| < R2500 | > R2500 | > R5000 |
| --- | --- | --- |
| > R10 000 | > R 20 000 | > R30 000 |

1. Government grants received – list how many recipients.

| **Child care** | **Care dependency** | **Disability** | **Old age** | **Other** |
| --- | --- | --- | --- | --- |
|  |  |  |  |  |

1. Where do you stay?

| INFORMAL SETTLEMENT | FORMAL DWELLING |
| --- | --- |

1. Please tell me more about your home?

| CORRUGATED IRON HOUSE (SHACK) | BRICK HOUSE | APARTMENT |
| --- | --- | --- |

1. How many adults live in the home where you live, including yourself?
2. How many children stay in this home__________________
3. Do you have the following amenities at home?

1. Electricity: **Y / N**

2. Flushing toilet/ pit toilet/ bucket system

3. Tap water inside home/ outside home/ no running water

1. **Residence status**
   1. Where does the mother of the patient originally come from?

|  | Mother | Father | Male partner | Caregiver 1 | Caregiver2 |
| --- | --- | --- | --- | --- | --- |
| SOUTH AFRICA |  |  |  |  |  |
| ZIMBABWE |  |  |  |  |  |
| MOZAMBIQUE |  |  |  |  |  |
| LESOTHO |  |  |  |  |  |
| SWAZILAND |  |  |  |  |  |
| OTHER (PLEASE NAME) |  |  |  |  |  |

What is the relationship of the caregiver to the patient (e.g.uncle, aunt)

Caregiver 1: ____________________

Caregiver 2: ____________________

**(IF BOTH PARENTS SOUTH AFRICAN CITIZENS PLEASE IGNORE QUESTIONS C-D and E)**

- 1. How long have you been staying in South Africa? MOTHER

FATHER

MALE PARTNER

- 1. What is your immigration status currently?

|  | Patient | Mother | Current Partner | Child | Caregiver 1 | Caregiver 2 |
| --- | --- | --- | --- | --- | --- | --- |
| PERMANENT RESIDENCE |  |  |  |  |  |  |
| TEMPORARY RESIDENCE |  |  |  |  |  |  |
| REFUGEE |  |  |  |  |  |  |
| NO LEGALISED STATUS |  |  |  |  |  |  |
| OTHER (PLEASE COMMENT) |  |  |  |  |  |  |

What is the relationship to the patient?

Caregiver 1: ____________________

Caregiver 2: ____________________

1. **Current admission:**
   1. When did this problem start? _______________________
   2. Who have you consulted before coming to hospital?

| TRADITIONAL HEALER | PRIVATE GP | CLINIC | OTHER HOSPITAL | NONE |
| --- | --- | --- | --- | --- |

- 1. If **none,** why did you come straight to hospital?

[Type a quote from the document or the summary of an interesting point. You can position the text box anywhere in the document. Use the Drawing Tools tab to change the formatting of the pull quote text box.]

- 1. How **many** times did you consult before you came to hospital?___________
  2. Why did you decide to come to **Kalafong Hospital**?

[Type a quote from the document or the summary of an interesting point. You can position the text box anywhere in the document. Use the Drawing Tools tab to change the formatting of the pull quote text box.]

- 1. Did you experience any problems when trying to access health care? **Y / N**
  2. If **Yes**, please explain?

[Type a quote from the document or the summary of an interesting point. You can position the text box anywhere in the document. Use the Drawing Tools tab to change the formatting of the pull quote text box.]

1. **Where has the patient been admitted to?**

| ICU/TRANSFER TO SBAH | SHORT STAY(WARD 8) | WARD 6 |
| --- | --- | --- |
| WARD 28 | WARD 27 | NICU |

1. Admission Severity Scale (Please tick the appropriate box)

| Lodger or normal neonate | Premature or Low birth weight **NOT** complicated | Premature or Low birth weight complicated |
| --- | --- | --- |
| Neonatal disease **NOT** severe | Disease needing ICU or NICU or death | Chronic disease, stable for investigation |
| Chronic disease with exacerbation | Non-severe disease or Short Stay (ward 8) admission | Acute/ Severe disease not needing ICU |

If needed please comment:

1. **Final diagnoses:**

| 1. |
| --- |
| 2. |
| 3. |
| 4. |
| 5. |

Appendix D- Master list/register of patients

| Nr. | Name and Surname | Hospital number |
| --- | --- | --- |
|  |  |  |
|  |  |  |
|  |  |  |
|  |  |  |
|  |  |  |
|  |  |  |
|  |  |  |
|  |  |  |
|  |  |  |
|  |  |  |
|  |  |  |
|  |  |  |
|  |  |  |
|  |  |  |
|  |  |  |
|  |  |  |
|  |  |  |
|  |  |  |
|  |  |  |
|  |  |  |
|  |  |  |
|  |  |  |
|  |  |  |
|  |  |  |
|  |  |  |
|  |  |  |
|  |  |  |
|  |  |  |
